# Fallacy of the Unique Genome: Sequence Diversity within Single *Helicobacter pylori* Strains

**DOI:** 10.1128/mBio.02321-16

**Published:** 2017-02-21

**Authors:** Jenny L. Draper, Lori M. Hansen, David L. Bernick, Samar Abedrabbo, Jason G. Underwood, Nguyet Kong, Bihua C. Huang, Allison M. Weis, Bart C. Weimer, Arnoud H. M. van Vliet, Nader Pourmand, Jay V. Solnick, Kevin Karplus, Karen M. Ottemann

**Affiliations:** a Institute of Environmental Science and Research, Porirua, New Zealand; b Department of Biomolecular Engineering, UC Santa Cruz, Santa Cruz, California, USA; c Department of Microbiology & Environmental Toxicology, UC Santa Cruz, Santa Cruz, California, USA; d Departments of Medicine and Microbiology & Immunology, Center for Comparative Medicine, UC Davis, California, USA; e Pacific Biosciences, Menlo Park, California, USA; f Department of Population Health and Reproduction, 100K Pathogen Genome Project, UC Davis, Davis, California, USA; g Department of Pathology and Infectious Diseases, School of Veterinary Medicine, Faculty of Health and Medical Sciences, University of Surrey, Guildford, United Kingdom; University of Maryland, School of Medicine

## Abstract

Many bacterial genomes are highly variable but nonetheless are typically published as a single assembled genome. Experiments tracking bacterial genome evolution have not looked at the variation present at a given point in time. Here, we analyzed the mouse-passaged *Helicobacter pylori* strain SS1 and its parent PMSS1 to assess intra- and intergenomic variability. Using high sequence coverage depth and experimental validation, we detected extensive genome plasticity within these *H. pylori* isolates, including movement of the transposable element IS*607*, large and small inversions, multiple single nucleotide polymorphisms, and variation in *cagA* copy number. The *cagA* gene was found as 1 to 4 tandem copies located off the *cag* island in both SS1 and PMSS1; this copy number variation correlated with protein expression. To gain insight into the changes that occurred during mouse adaptation, we also compared SS1 and PMSS1 and observed 46 differences that were distinct from the within-genome variation. The most substantial was an insertion in *cagY*, which encodes a protein required for a type IV secretion system function. We detected modifications in genes coding for two proteins known to affect mouse colonization, the HpaA neuraminyllactose-binding protein and the FutB α-1,3 lipopolysaccharide (LPS) fucosyltransferase, as well as genes predicted to modulate diverse properties. In sum, our work suggests that data from consensus genome assemblies from single colonies may be misleading by failing to represent the variability present. Furthermore, we show that high-depth genomic sequencing data of a population can be analyzed to gain insight into the normal variation within bacterial strains.

## IMPORTANCE

Although it is well known that many bacterial genomes are highly variable, it is nonetheless traditional to refer to, analyze, and publish “the genome” of a bacterial strain. Variability is usually reduced (“only sequence from a single colony”), ignored (“just publish the consensus”), or placed in the “too-hard” basket (“analysis of raw read data is more robust”). Now that whole-genome sequences are regularly used to assess virulence and track outbreaks, a better understanding of the baseline genomic variation present within single strains is needed. Here, we describe the variability seen in typical working stocks and colonies of pathogen *Helicobacter pylori* model strains SS1 and PMSS1 as revealed by use of high-coverage mate pair next-generation sequencing (NGS) and confirmed by traditional laboratory techniques. This work demonstrates that reliance on a consensus assembly as “the genome” of a bacterial strain may be misleading.

## INTRODUCTION

Sequencing of individual isolated bacterial genomes is now commonplace, with thousands being added to the public domain yearly. However, the typical sequencing and publishing procedures minimize or ignore the genomic variation present within bacterial isolates and continue to treat the genomes as relatively static, homogeneous entities. Laboratory experiments typically utilize bacterial strains—a defined subtype of a bacterial species—that were isolated as a single colony several generations prior to use. Although numerous studies have examined within-strain bacterial variability over time or in response to specific conditions, from early observations involving sectored colony formation (1) to recent work monitoring *E. coli* genome rearrangements over time (2), less is known about the extent of the genetic changes to expect within a typical laboratory culture (3). *H. pylori* is a microbe that is known to have a highly variable genome (4). *H. pylori* strains vary from each other by numerous gene rearrangements, inversions, sequence variation, and gene gain or loss (4, 5). Such changes have been observed to occur even within the same strain during the course of infection (5–7). The fluidity of the *H. pylori* genome is influenced by a preponderance of repeats, transposable elements, and restriction/modification (R/M) systems, combined with a lack of some typical DNA repair mechanism components (5, 8–16). The degree of genetic variation within *H. pylori* populations that are several generations removed from single-colony purification and maintained under typical laboratory conditions, however, is unknown.

*H. pylori* is highly adapted to humans, and strains capable of stably infecting mice are relatively rare (17). One such strain, named SS1, has become a field standard for mouse work (18). To isolate SS1, gastric homogenate obtained from a patient with gastric ulcers was plated onto *H. pylori* selective media to obtain single colonies, including one named strain 10700. These single colonies were subcultured several times to increase their numbers, mixed with human gastric biopsy specimen homogenates, and used to intragastrically infect mice. Strain 10700, now called PMSS1, for pre-mouse SS1 (19), was capable of long-term colonization in the mouse stomach (18). This strain was reisolated after mouse infection, representing a so-called “mouse passage,” as a single colony to create SS1. SS1 quickly became a standard for research into *H. pylori* pathogenicity and virulence. More recently, PMSS1, SS1’s parent, has become a popular experimental model strain because it has a greater ability to induce disease (19).

PMSS1 and SS1 both express the key *H. pylori* virulence factors: vacuolating cytotoxin (VacA) and CagA, the main product of the cytotoxin-associated pathogenicity island (*cag* PAI) type IV secretion system (T4SS). The *cag* PAI T4SS triggers inflammation on its own, presumably via interactions with the host cells, as well as via delivery of proinflammatory molecules, including CagA (20, 21). The SS1 *cag* PAI-harbored T4SS, however, is nonfunctional at least in part due to the presence of a defective CagY protein (22, 23). Loss of CagY creates strains that are unable to deliver *cag* PAI cargo and have minimal inflammatory ability. PMSS1, in contrast, has a fully functional *cag* PAI*-*harbored T4SS, consistent with its ability to cause severe disease in its original human host and in mice (18, 19). PMSS1 has therefore become a strain of choice for studying the function of the *H. pylori* T4SS in mice. However, the extent and nature of other genetic changes in PMSS1 that occurred during mouse passage transformation into SS1 have remained largely speculative. Therefore, in this study we set out to sequence this pair of genomes to achieve two objectives: (i) to gain an understanding of the genomic variation that exists within recent single-colony isolates of these strains and (ii) to identify the genomic changes that occurred during mouse-induced host adaptation and enable SS1 to thrive in this new host.

## RESULTS

### SS1 has a typical *H. pylori* genome.

We initiated this study to examine the variability within bacterial populations that were several generations removed from single-colony isolation using the *H. pylori* strain SS1. This strain has been used extensively within the field since its original isolation (18), so we hypothesized that its sequence would be of interest to many and would represent the scale of variation in such populations. For our study, we isolated genomic DNA (gDNA) from five plates of *H. pylori* SS1, growing as a lawn, to represent a normal working laboratory stock of *H. pylori*. This sample was referred to as the SS1 working stock population. The DNA was sequenced using SOLiD mate pair and 454 Titanium GS-FLX instruments to obtain >1,000- and 128-fold coverage, respectively. For the initial SS1 assembly, the 454 data were used to produce the main contigs using Newbler (now the Roche GS Assembler Software Package), and the SOLiD data were used to scaffold the contigs and to error-correct the 454 reads using custom Python scripts.

**FIG 1:**
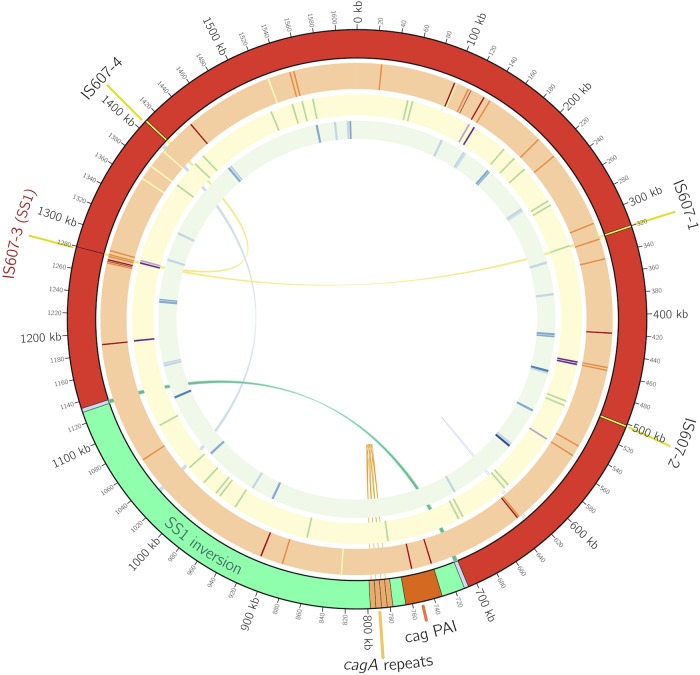
The SS1 and PMSS1 genomes display intragenomic variation. The data represent an overview of the variation observed in SS1 and PMSS1 in Circos format (59). The outer ring displays the key genomic features of the *H. pylori* SS1/PMSS1 genomes. The orange ring displays the PMSS1-SS1 differences as follows: yellow, difference in G/C homopolymer tract; orange, difference in A/T homopolymer tract; red, other difference. The yellow ring highlights disrupted genes: dark purple, disrupted only in SS1; light purple, disrupted only in PMSS1; green, disrupted in both SS1 and PMSS1. The green ring displays the putative observed SNPs with a frequency above 5% in SS1; the blue coloring darkens with increases in frequency from 5% to >50%. The inner links highlight the genomic rearrangements observed: the *cagA* repeat copy number variation (orange), the large SS1 inversion (green), two other putative observed inversions (light blue), and the movement of one of the IS*607* copies into site 3 in SS1 (yellow). Only features displayed on the outer ring are shown to scale. Features on the inner rings which would otherwise overlap have been shifted forward slightly so that all features are displayed.

The *H. pylori* SS1 genome had characteristics that were consistent with typical *H. pylori* genomes. The genome was ~1.6 Mb and had ~1,500 genes; we provide approximate numbers here because of variations in the copy numbers of several genes and insertion elements, as discussed in the next section (Fig. 1). The genome had ~39% G-C content. SS1 contained the previously reported plasmid pHPS1 (24).

To determine whether *H. pylori* SS1 has a typical complement of *H. pylori* genes, we compared the genes harbored in the SS1 genome to those harbored in the completed genomes of 41 *H. pylori* strains that span six *H. pylori* multilocus sequencing types (25), which contain 940 genes present in ≥95% of these strains. *H. pylori* SS1 was found to harbor intact versions of all but two of these. The missing genes encode the carbon starvation protein A (CstA) and l-lactate permease (LldP) (Table S1). Genes for these proteins are present in the SS1 genome but have alterations such that they appear to be pseudogenes as defined by having an open reading frame (ORF) that does not span the entire expected gene. PMSS1, in contrast, contains intact genes for these loci (see the “PMSS1” section below). The *lldP* alteration occurs in a homopolymeric tract, which might be attributed to 454 sequencing errors not corrected by the SOLiD reads; it is unknown, additionally, whether the encoded protein fragments could still have activity (Table S1). We also examined whether SS1 harbored any rare or unique genes, defined as those present in <15% or none of the 41 *H. pylori* genomes. These types of genes are also called “cloud” genes (26). We identified 17 such SS1 genes as well as the plasmid pHPS1 (Table S2). The majority of these genes are hypotheticals, so it is not yet clear what biological role they confer. Of note, although these genes are rare in the total *H. pylori* population, they were all genes found in at least one other non-SS1/PMSS1 *H. pylori* strain. This outcome suggests that SS1 does not have any recognizable genes to add to the *H. pylori* pan-genome.

10.1128/mBio.02321-16.4 TABLE S1  Core genes that are missing in the SS1 genome. Download TABLE S1, DOCX file, 0 MB. Copyright © 2017 Draper et al. 2017 Draper et al. This content is distributed under the terms of the Creative Commons Attribution 4.0 International license.

10.1128/mBio.02321-16.5 TABLE S2  Putative cloud genes of SS1. Download TABLE S2, DOCX file, 0.01 MB. Copyright © 2017 Draper et al. 2017 Draper et al. This content is distributed under the terms of the Creative Commons Attribution 4.0 International license.

### The sequenced SS1 working stock population shows substantial interpopulation variation.

The use of SOLiD mate pair sequencing and high depth of coverage with multiple technologies allowed us to observe that there was significant genome variation within the SS1 working stock population sequenced. This plasticity included movement of the transposon IS*607*, genomic inversions, variation in the *cagA* gene copy number, and potentially up to 58 single nucleotide polymorphisms (SNPs). Each of these variations is described below in more detail.

**FIG 2:**
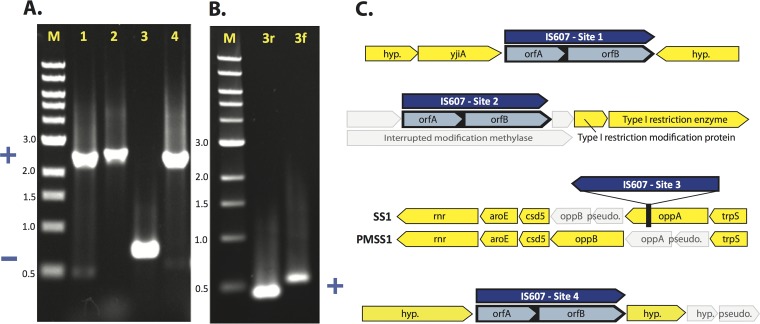
IS*607* is variably present at four sites in the SS1/PMSS1 genomes. (A) PCR performed on the sequenced SS1 genomic DNA sample, using primers flanking the four SS1 IS*607* insertion sites (lanes 1 to 4), showing larger bands where the site contains IS*607* (+) and smaller bands where the site does not contain IS*607* (-). M, 1-kb DNA marker. (B) PCR performed on the same genomic DNA sample, using primers located within IS*607* paired to the flanking (3f and 3r) primers for site 3, demonstrating that IS*607* is present at site 3 within the sequenced population, despite the predominance of the IS*607*(-) product seen using the flanking primers alone. (C) The genomic context of the four IS*607* insertion sites in SS1/PMSS1. IS*607* is shown in blue; disrupted and presumably nonfunctional genes are shown in gray. Sites 1 and 4 are not always occupied in SS1, but site 2, which disrupts an R/M system, is invariably occupied. The genomic contexts of the insertion sites are identical for SS1 and PMSS1 except for SS1 insertion site 3. At site 3, IS*607* is inserted into the otherwise intact *oppA* gene in a small subpopulation of SS1; no such insertions were observed in PMSS1, although the *oppA* gene is already disrupted in PMSS1. hyp., hypothetical gene; pseudo., pseudogene.

There was SS1 working stock population heterogeneity in the position of the IS*607* bacterial insertion element. IS*607* was previously found to exist in approximately one-fifth of *H. pylori* strains of a diverse collection using PCR analysis (10). It is reportedly present in most complete *H. pylori* genomes only once per genome, although it is found in strains B012A and B013A in 9 and 4 copies, respectively (7, 27). In SS1, IS*607* was present at three or four positions (Fig. 1 and 2). In addition to the presence of multiple copies of IS*607*, both the 454 and SOLiD mate pair sequencing results indicated heterogeneity in the working stock population, with all but one of the IS*607* sites being occupied in only part of the population. Targeted PCR using primers flanking the four IS*607* insertion sites confirmed that one site, so-called site 2 (Fig. 2), was always IS*607* positive, and that the others produced bands consistent with both IS*607*-positive and IS*607*-negative states (Fig. 2). The third site appeared to be predominantly in the IS*607*-negative state, possibly because it represents a recent transposition event. For this last insertion site, a PCR amplification using primers inside the IS*607* sequence confirmed that both IS*607*-negative and IS*607*-positive subpopulations existed within the SS1 working stock population. The IS*607* copies disrupted several genes. The permanent IS*607* insertion site disrupted a R/M system, two variable sites lay between uncharacterized genes, and the third variable insertion site disrupted the *oppA* gene (Fig. 2). These data suggest that IS*607* is mobile in the SS1 genome.

**FIG 3:**
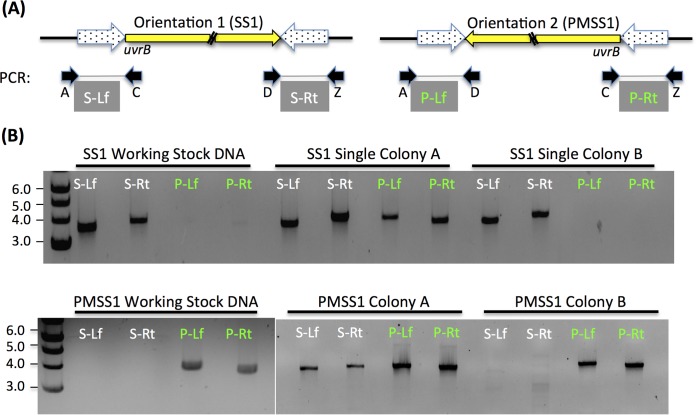
A large region variably inverts in SS1 and PMSS1. (A) Diagram showing the orientation of the large inversion region as a yellow arrow. Its size is 425,787 bp. The inversion that was most common in SS1 is shown on the left (Orientation 1), while that in PMSS1 is shown on the right (Orientation 2). The *uvrB* gene is within the inverted region, just inside one of the inverted repeats (dotted arrows). Primers to confirm the inversion orientation are labeled below each image as A, C, D, and Z. PCR amplification with these yields products A to C (S-Lf) and D to Z (S-Rt) in prientation 1/SS1 and products from A to D (P-Lf) and C to Z (P-Rt) in orientation 2/PMSS1. (B) Gels of PCR amplification products from the primer sets indicated in panel A with template DNA prepared from our working stock *H. pylori* or from single colonies that were isolated from the freezer stocks and subcultured once prior to DNA extraction. Six single colonies were isolated from each strain, and two representative colonies are shown.

Another striking variation within SS1 was a large inversion spanning roughly one-quarter of the genome (Fig. 1). The inverted region had one orientation in SS1 (called the SS1 orientation) and the opposite orientation in PMSS1 (Fig. 3A). Although the so-called SS1 orientation was found in the majority of the sequenced SS1 working stock population, it was not fixed, and we found evidence in the SOLiD sequencing data indicating that the working stock population possessed the inversion in both the SS1 orientation and the PMSS1 orientation. We further confirmed that both orientations existed using targeted PCR amplification followed by Sanger sequencing of data from the SS1 working stock population as well as from individual single colonies isolated from the stock population (Fig. 3B). We found that the working stocks were dominated by one orientation, although a faint band was detected for the PMSS1 orientation in the SS1 working stock DNA (Fig. 3B). Both inversion orientations were readily apparent in single colonies isolated from our stocks, likely because the percentage of the population that contained the nonparent inversion increased during this isolation (Fig. 3B). This inversion occurs between two inverted repeats which consist of three ORFs of unknown function; the third ORF is disrupted in one copy of the repeat. Evidence for two other inversions at separate sites was observed in the SS1 working stock population, based on SOLiD mate pair data. These consisted of an inversion of the *dcuA* and *ansA* genes that did not disrupt either coding sequence (CDS) and an inversion of a large region between the two copies of the 23S rRNA gene (Fig. 1). Taken together, these analyses suggest that regions of the SS1 genome frequently invert.

We also examined SNPs within the sequenced SS1 working stock population. To identify those that were reliably present, we analyzed the high-coverage SOLiD mate pair data for SNPs that met a threshold cutoff that was determined by producing a scatter diagram of numbers of duplicated mate pairs (*r*) versus percent support (Fig. S1). Reliable SNPs were found in at least 100 e^[0.23 (13-r)]^% of the deduplicated mate pairs, with at least *r* mate pairs supporting the minority position, where the exponential results from a straight dividing line on the semilog plot that spread out the scatter diagram most cleanly (Fig. S1). In essence, this means that, for example, 13 reads would be enough if they provided 100% support, but 33 reads would be needed for 1% support. For this analysis, reads were “deduplicated” by replacing multiple mate pairs that had identical color sequences for the two reads with a single mate pair, to minimize the effect of PCR errors on SNP calling. SNPs with low numbers of supporting reads (<30) were further filtered to accept only those with reads that spanned the entire SNP, not just the last few positions of a read. Using this conservative approach, we found 58 SNPs that occurred throughout the genome of the working stock population (Table 1 and Fig. 1). Of these, 12 were in intergenic regions and the remaining 46 were within predicted coding regions. Of those within coding regions, 14 were synonymous and 32 were missense or nonsynonymous. We confirmed that this approach had found true SNPs by PCR amplification and sequencing of an SNP in the *flhB* gene (Fig. S2). Both SNP variants were detected in the working stock population. The high-coverage sequence depth thus allowed detection of intragenome SNPs.

10.1128/mBio.02321-16.1 FIG S1  Analysis of SNP support. Scatter diagram with *y* axis data representing the numbers of duplicated mate pairs *r* and *x* axis data representing percent support. A straight dividing line on the semilog plot that spread out the scatter diagram points most cleanly gave rise to the exponential, which in turn was used to determine reliable SNPs as those in at least 100^e[0.23 (13-*r*)]^% of the deduplicated mate pairs, with at least *r* mate pairs supporting the minority position. Download FIG S1, TIF file, 2.2 MB. Copyright © 2017 Draper et al. 2017 Draper et al. This content is distributed under the terms of the Creative Commons Attribution 4.0 International license.

10.1128/mBio.02321-16.2 FIG S2  Analysis of the *flhB* (HPYLSS1_00566) region in SS1. (A) The two variants of the SNP, one of which causes a frameshift in the open reading frame. (B) Sanger sequencing results from PCR products of the region shown in panel A. The arrow indicates the variable SNP position. Download FIG S2, TIF file, 25.1 MB. Copyright © 2017 Draper et al. 2017 Draper et al. This content is distributed under the terms of the Creative Commons Attribution 4.0 International license.

10.1128/mBio.02321-16.3 FIG S3  Multiple alignment of the *cagA* gene region in SS1 with the currently available complete *H. pylori* genomes containing a similar off-island *cagA* location. Genomes from *H. pylori* strains B8, ELS37, HUP-B14, J166, and NY40 were obtained from NCBI and aligned using the Mauve multiple genome alignment algorithm in Geneious (57). The top bar shows overall percent identity of the multiple alignment. Gray bars represent each genome, with the black vertical lines in the bar indicating nucleotides that differ and thin horizontal lines representing gaps in the alignment. Predicted coding sequences are shown in yellow, and the ~6-kb *cagA* repeat region is highlighted in blue. For the purposes of this alignment, the SS1 *cagA* gene is shown as a single copy. The distance between the rest of the *cag* PAI and the start of *cagA* is roughly 15 kb. Download FIG S3, TIF file, 22.2 MB. Copyright © 2017 Draper et al. 2017 Draper et al. This content is distributed under the terms of the Creative Commons Attribution 4.0 International license.

**TABLE 1:** Intragenomic SNPs within the sequenced SS1 population^a^

Position	Base 1 (no. of reads)	Base 2 (no. of reads)	% minority SNP	Locus	Gene or product	Effect	Homopolymer
114146	A (387)	T (27)	6.52	HPYLSS1_00109	hyp.	Nonsynonymous F:L	A
147049	T (436)	A (41)	8.60	HPYLSS1_00135	*fixN*	Synonymous	
148431	A (305)	C (26)	7.85	HPYLSS1_00136	*fixO*	Synonymous	
186359	T (221)	C (46)	17.23	HPYLSS1_00179	*clsA*	Synonymous	
187828	T (269)	G (48)	15.14	HPYLSS1_00181	*frdA*	Nonsynonymous K:N	
252785	A (504)	T (37)	6.84	HPYLSS1_00242	omp	Nonsynonymous Q:H	
252826	T (1,114)	A (30)	2.62	HPYLSS1_00242	omp	Missense	
253141	A (430)	T (56)	11.52	HPYLSS1_00243	omp	Nonsynonymous K:I	A
254527	T (397)	Del (39)	8.94	Intergenic			
329785	A (683)	T (38)	5.27	HPYLSS1_00312	*flgH*	Nonsynonymous N:Y	
329895	A (307)	G (62)	16.80	HPYLSS1_00312	*flgH*	Synonymous	A
372026	A (589)	T (45)	7.10	HPYLSS1_00353	hyp.	Nonsynonymous N:Y	
424362	T (247)	A (41)	14.24	HPYLSS1_00411	Response regulator	Synonymous	
426404	GTT (265)	CTC (67)	20.18	HPYLSS1_00412	*ispDF*	Nonsynonymous K:R	T
472864	T (495)	A (175)	26.12	HPYLSS1_00456	NYN domain	Nonsynonymous K:I	
474596	A (557)	T (43)	7.17	HPYLSS1_00458	*tdhA*	Nonsynonymous L:I	
528077	A (196)	C (29)	12.89	HPYLSS1_00513	*hom*	Nonsynonymous F:C	
578269	A (572)	C (27)	4.51	HPYLSS1_00562	*tyrS*	Nonsynonymous K:Q	A
579815	T (588)	A (58)	8.98	HPYLSS1_00563	*pyrD_2*	Nonsynonymous F:I	
581267	T (330)	Del (29)	8.08	HPYLSS1_00565	hyp.	Missense	T
582628	C (4)	A (18)	81.82	HPYLSS1_00566	*flhB*	Missense	C
675623	T (948)	A (42)	4.24	HPYLSS1_00648	*uvrD*	Nonsynonymous N:Y	T
676491	T (400)	G (35)	8.05	HPYLSS1_00649	*taqIM*	Nonsynonymous Q:H	
702344	A (688)	G (35)	4.84	Intergenic			
731566	T (6)	Del (15)	28.57	Intergenic			
818638	T (402)	C (69)	14.65	HPYLSS1_00957	*ppiD*	Synonymous	
818676	A (430)	G (49)	10.23	HPYLSS1_00957	*ppiD*	Nonsynonymous F:L	
889231	T (281)	A (27)	8.77	Intergenic			T
897376	T (144)	Del (45)	23.81	Intergenic			T
918653	A (213)	C (32)	13.06	HPYLSS1_00869	*rnc*	Nonsynonymous F:L	
1152909	T (784)	G (33)	4.04	HPYLSS1_01082	*parB*	Nonsynonymous N:H	
1153593	T (785)	A (35)	4.27	HPYLSS1_01082	*parB*	Nonsynonymous N:Y	T
1153634	T (755)	A (71)	8.60	HPYLSS1_01083	*soj*	Nonsynonymous K:I	T
1155502	A (509)	G (38)	6.95	HPYLSS1_01085	*fmt*	Synonymous	A
1198398	T (260)	Del (30)	10.34	HPYLSS1_01122	omp	Missense	T
1205511	A (628)	G (28)	4.27	HPYLSS1_01129	Na exchanger	Nonsynonymous F:L	A
1237191	T (222)	A (41)	15.59	Intergenic			
1239091	A (287)	T (82)	22.22	Intergenic			
1293001	T (269)	A (29)	9.73	HPYLSS1_01216	*nuoH*	Synonymous	
1329559	T (672)	Del (34)	4.8	HPYLSS1_01263	*rplV*	Missense	T
1329936	T (570)	G (46)	7.47	HPYLSS1_01264	*rpsS*	Nonsynonymous Q:H	
1331340	T (1,505)	C (35)	2.27	Intergenic			
1331598	T (838)	A (43)	4.88	HPYLSS1_01267	*rplD*	Missense	
1431054	A (175)	Del (37)	17.45	Intergenic			A
1437973	A (418)	G (62)	12.92	Intergenic			
1438641	T (1,214)	Del (139)	10.27	Intergenic			T
1438946	A (559)	G (38)	6.37	HPYLSS1_01366	hyp.	Nonsynonymous F:S	
1440337	T (240)	A (60)	20.00	HPYLSS1_01368	DHH family	Synonymous	
1510384	T (531)	C (27)	4.84	HPYLSS1_01436	*plsY*	Synonymous	
1511744	T (1,091)	A (37)	3.28	HPYLSS1_01439	omp	Nonsynonymous L:I	
1512023	A (1,235)	T (45)	3.52	HPYLSS1_01439	omp	Nonsynonymous N:Y	
1512054	A (1,012)	Del (35)	3.3	HPYLSS1_01439	omp	Missense	A
1566497	T (190)	C (23)	10.80	HPYLSS1_01480	MTase	Synonymous	
1589945	T (282)	Del (26)	8.4	Intergenic			T
1590568	A (340)	T (37)	9.81	HPYLSS1_01500	*penA*	Nonsynonymous L:I	
1607348	T (943)	A (90)	8.71	HPYLSS1_01519	*ribE*	Synonymous	
1609179	T (1,207)	C (34)	2.74	HPYLSS1_01522	*metI*	Synonymous	
1609811	T (253)	A (39)	13.36	HPYLSS1_01523	hyp.	Synonymous	

a Base 1 and Base 2 indicate the two distinct bases, with the number of deduplicated SOLiD sequencing reads supporting each call indicated in parentheses. The data in the “Homopolymer” column indicate whether the site is part of a homopolymer tract of length 3 or greater. The data in the “Gene or product” column indicate whether the SNP is intergenic or within a gene and the putative identity or function of the gene. hyp., hypothetical; omp, putative outer membrane protein.

### *cagA* copy numbers are highly unstable in SS1.

**FIG 4:**
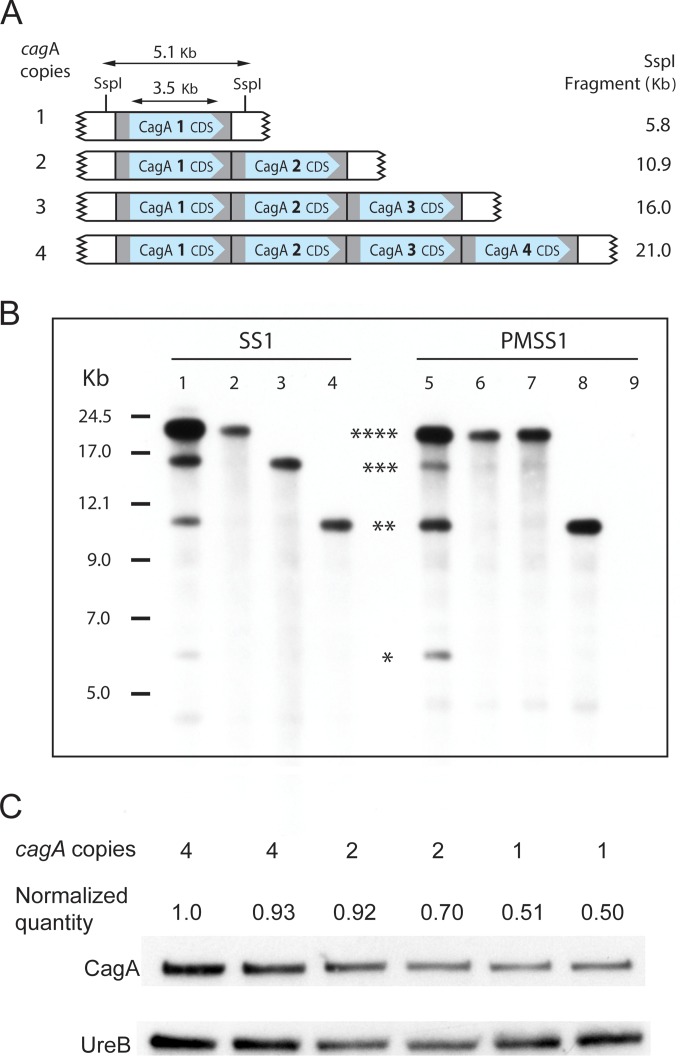
*H. pylori* SS1 and PMSS1 are gene copy number variable at the *cagA* locus. (A) Schematic diagram showing tandem arrays of the identical 5,072-bp repeat regions at the *cagA* locus, all of which contain identical copies of the 3,540-bp *cagA* gene. The SspI fragment sizes of strains with 1 to 4 *cagA* copies are shown at the right. The drawing is not to scale. (B) Southern blot of SspI-digested genomic DNA from *H. pylori* SS1 or PMSS1 probed with a 297-bp PCR product amplified from SS1 *cagA* bp 1217 to 1514. The original working stocks of SS1 (lane 1) and PMSS1 (lane 5) showed bands corresponding in size to 4, 3, 2, and 1 copies of *cagA* (asterisks). Subculture of 4 single colonies from the freezer stocks demonstrated clones with 4, 3, or 2 copies of *cagA* (lanes 2 to 4) from SS1; subculture of 12 single colonies showed either 4 or 2 copies of *cagA* in PMSS1 (lanes 6 to 8). PMSS1 with a *cagA* deletion served as a negative control (lane 9). A kilobase ladder is shown at the left. (C) Western blot of *H. pylori* PMSS1 to examine whether the *cagA* copy number is positively correlated with protein expression. For this analysis, six individual single-colony isolates of PMSS1 were used, two each with four copies, two copies, or one copy of *cagA*. Relative quantities of protein in each band were determined using Image Lab software (Bio-Rad Laboratories). The density of the CagA band was divided by the density of the corresponding UreB band to obtain the normalized quantity of CagA to account for differences in gel loading.

During our initial assembly of SS1, we observed an overabundance of sequencing reads in the *cagA* region and mate pairs mapping from *cagA* to *cagA*. Targeted PCR and Sanger sequencing (not shown) confirmed that there was at least one tandem duplication of the *cagA* gene. However, the sequence coverage spike over *cagA* suggested there could be three or more *cagA* copies arranged in a tandem repeat. We could not determine a precise copy number or assess potential subpopulation variability from the DNA sequencing data. Therefore, we performed Southern blotting to determine the *cagA* copy number. Analysis of SS1 parent strains showed bands that corresponded in size to 1 to 4 copies of *cagA* (Fig. 4A). Subculture of individual colonies from these parent cultures demonstrated isolates containing between one and four copies of *cagA* (Fig. 4B). Similar results were obtained with PMSS1 and are described in the “PMSS1” section below. These observations suggested that *cagA* copy numbers are highly unstable in both SS1 and PMSS1. Western blot analysis demonstrated that increased *cagA* copy numbers correlated with increased CagA protein levels (Fig. 4C). Our data support the conclusion that SS1 contains between 1 and 4 copies of the *cagA* gene.

**FIG 5:**
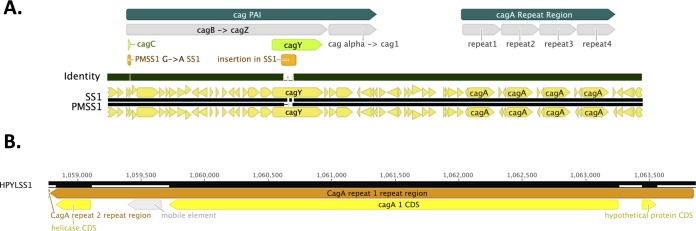
The *cag* PAI and *cagA* regions possess only two sequence differences between SS1 and PMSS1. (A) Diagram shows an alignment of the *cag* PAI and *cagA* regions of SS1 and PMSS1, with a dark green middle “identity” bar indicating their genetic identity. The two regions that differ—in *cagY* and *cagC*—are indicated and detailed in Table 2. (B) Diagram showing a single *cagA* repeat, with the *cagA* gene, promoter region, and two open reading frames (hypothetical and helicase).

We additionally noted that the SS1 *cagA* copies were “off island,” meaning that they were separated from the other genes of the *cag* PAI, in a region flanked by genes encoding “glutamate racemase” (*murI*) and a “hypothetical protein” (Fig. 5). This genomic organization is also found in several other strains, although those genomes reportedly have only one copy of *cagA* (Fig. S2). The space separating the genes in each *cagA* repeat was relatively small and contained the *cagA* promoter as well as a couple of small predicted ORFs that are not typically found in the *cag* PAI and may have been pseudogene remnants of the upstream *murI* gene. It was not possible to reliably determine whether these tandem repeats of ~6 kb each were 100% identical using only next-generation sequencing technologies, especially given the evident rate of recombination occurring between the loci.

### There are few coding sequence differences between SS1 and PMSS1.

Given the variability that we observed within the SS1 working stock population, we next asked how much variation existed between SS1 and its parent PMSS1, as well as within a PMSS1 working stock population. We sequenced a sample of PMSS1 that was prepared similarly to the way SS1 was prepared, using Pacific Biosciences long-read technology with 586-fold coverage, supplemented with Illumina short-read technology data from related mouse-passaged isolates for error correction (described in Materials and Methods [“Corrections to genomes”]). Overall, the SS1 and PMSS1 genomes were highly similar, with 99.9% identity (Fig. 1). We identified 45 differences between the genomes and 1 difference on the plasmid, 28 of which were in RNA or protein coding regions (Table 2) and 18 of which were in intergenic regions (Table S3). These coding region insertions and deletions (indels) and SNPs mapped to 23 coding sequences (Table 2). The affected genes spanned a range of categories, as described below.

10.1128/mBio.02321-16.6 TABLE S3  SS1-PMSS1 differences that were in intergenic regions. Download TABLE S3, DOCX file, 0 MB. Copyright © 2017 Draper et al. 2017 Draper et al. This content is distributed under the terms of the Creative Commons Attribution 4.0 International license.

**TABLE 2:** Differences between SS1 and PMSS1^a^

PMSS1 locus/category	Gene	26695 ortholog	Putative function	Start (PMSS1)	Difference (SS1:PMSS1)	Effect of alteration	Homopolymer
Pathogenesis							
HPYLPMSS1_00538	*hpaA*	HP0797	N-Acetylneuraminyllactose binding hemagglutinin precursor	549911	A:del	Disruption in PMSS1 (truncation)	A
HPYLPMSS1_00703	*cagC*	HP0546	cag T4SS (Cag25)	733073	A:G	Nonsynonymous PMSS1:SS1 A34:T	
HPYLPMSS1_00721	*cagY*	HP0527	cag T4SS (Cag27)	753548	CGTA:TGTT	Disruption in SS1	
HPYLPMSS1_00721	*cagY*	HP0527	cag T4SS (Cag27)	753590	Large indel	Gene lengthened in SS1	
HPYLPMSS1_00721	*cagY*	HP0527	cag T4SS (Cag27)	753527	T:C	Disruption in SS1	
Transport							
HPYLPMSS1_00124	*sdaC*	HP0133	Serine transporter	134529	G:A	Disruption in PMSS1	
HPYLPMSS1_00131	*lldP_1*	HP1040	Lactate transporter	141372	T:del	Disruption in SS1 (truncation)	T
HPYLPMSS1_00437	*modD*	HP0475	Mo transport (putative)	454124	A:del	Disruption in SS1 (truncation)	A
HPYLPMSS1_00619	*argO*	HP0718	Arginine export	634127	G:A	Nonsynonymous PMSS1:SS1 562I:V	
HPYLPMSS1_01114	*cstA*	HP1168	Peptide transport	1188475	A:G	Disruption in SS1	
HPYLPMSS1_01200	*oppB*	HP1251	Peptide transport	1278074	A:del	Disruption in SS1	A
HPYLPMSS1_01201	*oppA*	HP1252	Peptide transport	1279518	T:del	Disruption in PMSS1	T
Motility/chemotaxis							
HPYLPMSS1_00098	*tlpB*	HP0103	Chemoreceptor (pH, autoinducer-2, and urea)	101463	T:C	Nonsynonymous PMSS1:SS1 R443:H	
Regulation							
HPYLPMSS1_004057	*fur*	HP1027	Transcriptional regulator	419034	G:T	Nonsynonymous PMSS1:SS1 H45:P	
LPS and surface							
HPYLPMSS1_00858	*futB*	HP0651	LPS modification	9082801	168-bp deletion in SS1	Variation in heptad repeat no.	
HPYLPMSS1_00439	*omp*	HP0462	Outer membrane protein	456915	Del:A	Disruption in SS1	A
DNA and RNA							
HPYLPMSS1_01194	*ssb*	HP1245	Single-stranded binding protein	1272174	Multiple SNPs	Nonsynonymous PMSS1:SS1 PSYAQNS:QSYPQNA	
HPYLPMSS1_01301		HP1354/55	DNA methylase	1363869	G:del	Gene shorter in SS1	G
HPYLPMSS1_01313		HP1369	Adenine-specific DNA methylase	1379237	G:del	Disruption in SS1 and PMSS1	G
HPYLPMSS1_01365		None	16S rRNA	1437353	A:G	Noncoding RNA gene	
Unknown function							
HPYLPMSS1_01207		HP1258	Rdd family protein	1284064	Del:A	Gene shorter in SS1	A
HPYLPMSS1_01449		None	Hypothetical	1528396	C:del	Gene shorter in PMSS1	C
Synonymous							
HPYLPMSS1_00721	*cagY*	HP0527	cag T4SS (Cag27)	753548	CGTA:TGTT	Synonymous	
HPYLPMSS1_00721	*cagY*	HP0527	cag T4SS (Cag27)	753527	T:C	Synonymous	
HPYLPMSS1_00111	*topA*	HP0444	DNA topoisomerase I	118799	C:T	Synonymous	
HPYLPMSS1_00111	*topA*	HP0444	DNA topoisomerase I	117551	C:T	Synonymous	
HPYLPMSS1_00111	*topA*	HP0444	DNA topoisomerase I	117563	G·C	Synonymous	
HPYLPMSS1_00111	*topA*	HP0444	DNA topoisomerase I	117182	T:C	Synonymous	
HPYLPMSS1_01194	*ssb*	HP1245	Single-stranded binding protein	1272229	G:A	Synonymous	
HPYLPMSS1_01197	*rnr*	HP1248	RNase R	1274656	A:G	Synonymous	

a The difference column lists the sequence in SS1 and then that in PMSS1. del, no base present. The data in the “Homopolymer” column indicate whether the alteration occurred within a homopolymer tract of length 3 or greater and what base comprised the tract.

In the *cag* PAI, PMSS1 and SS1 differed in only two genes, *cagY* and *cagC* (Fig. 5 and Table 2). Both of these encode proteins that are required for *cag* T4SS function but dispensable for pilus formation (22, 28). These mutations are consistent with the observation that SS1 has a nonfunctional *cag* PAI T4SS that cannot deliver CagA effectively, while PMSS1 maintains a functional *cag* PAI (22, 23, 29, 30). CagY is a key *cag* PAI protein that is under high selective pressure via the mammalian immune system and is frequently altered in mouse-infecting strains (22). We observed that, consistent with these observations, *cagY* was highly altered between PMSS1 and SS1, bearing three distinct differences (Table 2). Comparing *cagY* between PMSS1 and SS1, we identified a 4-bp gene replacement, one SNP, and one 50-bp region differing in length and sequence (Table 2 and Fig. 5), similar to changes reported previously (22). *cagC*, on the other hand, had only a single SNP which encoded an amino acid substitution at position 35 of alanine for threonine. The effect of this CagC alteration is unknown, though it could, in principle, explain why replacement of *cagY* from SS1 with that from PMSS1 does not fully restore T4SS function (22). Beyond *cagY* and *cagC*, the entire *cag* PAI regions of SS1 and PMSS1 were identical (Fig. 5). Thus, the *cag* PAI region shows evidence of selection during mouse passage, but only at *cagY* and, to a lesser degree, *cagC*.

SS1 contained two other variations compared to PMSS1 in known mouse colonization or pathogenicity factors: the HpaA neuraminyllactose-binding protein and the FucT α-1,3 lipopolysaccharide (LPS) fucosyltransferase (Table 2). HpaA is essential for colonization of mice by SS1 (31). In line with this idea, we found that the *hpaA* gene is intact in SS1 but is disrupted by a frameshift in PMSS1. *hpaA* encodes a lipoprotein that binds neuraminyllactose, but its exact role in pathogenesis is unknown (31, 32).

*H. pylori* SS1 and PMSS1 encode two FucT α-1,3 fucosyltransferases, FutA and FutB. These enzymes add Lewis X sugars to the O antigen of *H. pylori* LPS. FutA and FutB enzymes contain a variation in a particular heptad repeat region that places the active sites various distances from the bacterial surface (33, 34). We observed that *futB* of PMSS1 encoded 12 amino acid heptad repeats, while the corresponding paralog of SS1 contained 4.

The biological effects of other differences between SS1 and PMSS1 are unclear (Table 2). SS1 may possibly demonstrate less signaling from the chemoreceptor TlpB than does PMSS1 due to a mutation that affects a conserved signaling residue. SS1 may also have an increased ability to transport serine due to restoration of full-length *sdaC*. Although there are other alterations in genes involved in various cellular processes, there is not enough information about the effects of the polymorphisms to predict how they would change any phenotypes.

### PMSS1 shows working stock population variability similar to that shown by SS1, including at *cagA.*

Our results demonstrated that SS1 has substantial working stock population variability, so we assessed whether PMSS1 would show a similar degree of variability. We focused on the insertion elements, inversions, and copy number alterations but did not analyze SNPs because the Pacbio sequencing technology used for PMSS1 has a high error rate and thus did not allow confident SNP detection. IS*607* was not seen at the rare third site in PMSS1, suggesting that this was a recent transposition event. At the large inverted region, we found that PMSS1 contained this inverted region in the orientation opposite to that seen in SS1 (Fig. 3). However, as seen with SS1, we were able to detect both orientations within the PMSS1 working stock population via PCR (Fig. 3). Last, we detected a variation in the *cagA* copy numbers of 1 to 4 that was similar to that observed for SS1 (Fig. 4). Overall, this analysis suggests that SS1 and PMSS1 display similar levels of intragenome variability.

## DISCUSSION

We report here the genomic sequences of *H. pylori* strains SS1 and PMSS1 and observe that, within laboratory working stocks and even freshly isolated single colonies, there was not one constant genome for each isolate. Instead, we found that there was rather a collection of genomes that differed with regard to gene copy number, insertion sequences, inversions, and SNPs. These variations resulted in a group of bacteria that likely possess different phenotypes with regard to multiple processes. These working stock population differences highlight and support the concept that *H. pylori* genomes are dynamic during laboratory culture and that this heterogeneity can be detected using high-depth sequence coverage that incorporates long-read or mate pair technologies.

The substantial intragenomic variation observed in this study raised the issue of how to report “the” SS1 or PMSS1 genome. For example, should one, two, three, or four copies of *cagA* be included, and should three or four copies of IS*607* be included? Due to the mixed nature of our sequenced population, the association of individual combinations of variants could not be determined, ruling out publication of multiple genomes. We therefore chose to take the traditional approach of publishing a consensus genome containing the most common variant but augmented this information by including substantial annotation to indicate the variability that we observed.

Detection of genomic rearrangements in subpopulations of sequence data using NGS technologies requires use of long reads or mate pairs of high coverage depth, as well as assembly algorithms that specifically look for and report rearrangements. In this study, the SOLiD mate pairs for SS1 performed this role, using custom scripts to detect the rearrangements. Detecting intrapopulation SNPs also requires high coverage depth with a technology that has a low error rate; here, we used SOLiD for this, with the added caveat of ignoring all duplicated reads to avoid the potential for counting PCR errors as SNPs. MiSeq or HiSeq technologies would also work for this purpose, but only at coverage levels well above 100×. Traditionally, intrapopulation variation within bacterial genome sequence data has been ignored due to the difficulty of determining whether such variations represent “real” variations or sequencing errors. We hope that in the future more work will be dedicated to developing robust methods for reliably detecting intrapopulation variation within bacterial whole-genome sequence datasets.

Most of the within-strain variation that we detected appeared to be highly mutable, occurring even in isolates that were recently single colonies. For example, we detected both orientations of the large inversion in both SS1 and PMSS1 in isolates from recent single colonies. We also found single-colony isolates containing *cagA* repeat arrays ranging from 1 to 4 copies in number, with some appearing to have internal variation. We furthermore observed that infection of a mouse with a strain bearing one *cagA* copy would result in isolates with other copy numbers (data not shown). Studies suggest that these phenotypes are not unique to SS1. SS1 has a mutation rate similar to the rates seen with other *H. pylori* strains (35). Furthermore, Jang et al. report that, in work done simultaneously with this work, diverse *H. pylori* strains were found to have multiple copies of the *cagA* gene (60). These studies suggest that SS1 and PMSS1 are not unique in their variation and, furthermore, that even an isolate from a single colony contains genomic variation that is detectable using high-depth sequencing.

Given the within-strain variation, we were surprised that there were relatively few differences between PMSS1 and SS1. In >1.6 million bp, there were only 46 points of difference (Tables 2 and S^3^). This finding is consistent with the idea that PMSS1 is actually already highly optimized for mouse colonization, requiring only modest changes to enhance this property (18, 19). The PMSS1-SS1 differences were found in genes encoding proteins covering a range of metabolic properties, although many appeared to be in transporters. Heithoff and colleagues reported that diverse metabolic changes are needed for *Salmonella* to become better able to colonize mice (36), suggesting that broad metabolic adaptation is a common strategy associated with host adaptation.

We expected changes in the *cag* PAI between PMSS1 and SS1 given that it was already well known that SS1 had lost Cag T4SS functionality (22, 23, 37). Surprisingly, there were only two genes changed between the *cag* PAIs: multiple mutations in the *cagY* gene, including a large indel noted previously (22), and a single nucleotide SNP in *cagC* (Fig. 5 and Table 2). Otherwise, the entire 33-kb islands in SS1 and PMSS1 were identical. Another substantial SS1-PMSS1 difference was related to LPS fucosylation, suggesting that LPS modification may be important for colonization of the mouse stomach. Indeed, variations in the expression and number of heptad repeats in FutA and FutB occur during animal and human infection, but the functional consequence of this variation is not yet known (33, 38). Some of the genes that we observed to be changed here were also seen to undergo selection in other analyses of *H. pylori* during mouse selection. For example, *hpaA* was one of 23 genes that increased in expression after *H. pylori* strains were subjected to mouse passage (39).

We report here that SS1 and PMSS1 have multiple copies of the *cagA* gene, a finding similar to that reported in the companion manuscript (60). CagA is a critical multifunctional virulence factor of *H. pylori* (21). CagA-positive *H. pylori* strains cause more-severe inflammation, ulcers, and cancer. Indeed, CagA is considered a bona fide bacterial oncogene, because ectopic expression of it promotes gastric cancer (40). Thus, the observation that *H. pylori* can exhibit variations in *cagA* copy numbers and CagA protein levels is highly significant. The mechanism for *cagA* copy number variation, however, is unknown. There are two Amerindian *H. pylori* strains with more than one *cagA* gene, called Shi470 and V225d (41, 42). The types of copy number variations, however, differ from what we observed here. In those cases, there were two *cagA* copies, with the second copy of *cagA* and a second *cagB* inserted in the middle of the *cag* PAI, between *cagQ/cag14* and *cagP/cag15* (41, 42). In one case, the encoded *cagA* appears to be a pseudogene, based on insertion mutations that alter the reading frame (41). In our work, *cagA* was the only genomic region that underwent duplication. It therefore seems possible that there is a molecular mechanism allowing specific gene amplification (43). We did identify some repeated sequences in the form of mini-IS*605* sequences flanking the *cagA* genes. Mini-IS*605* sequences have been reported previously for portions of the *cag* island, including near *cagA*, but these sequences are found in strains that do not amplify this gene, so it is not clear what role the mini-IS*605* plays (44, 45). Indeed, given that SS1 expresses but does not deliver CagA effectively, it is somewhat puzzling why the *cagA* copy numbers would vary in this strain. We speculate that either CagA serves a role that is independent of *cag* T4SS function or that the duplications served a function in PMSS1 and have been retained in SS1. Future experimentation is required to dissect these possibilities. We also noted that the *cagA* copies are placed at a distance from the other genes of the *cag* PAI. This off-island arrangement is seen in a few other complete *H. pylori* genomes (B8, HUP-B14, J166, NY40), but these strains have not been reported to contain more than one copy of *cagA*. A survey of MiSeq data from three isolates of one such strain, J166, showed no coverage spikes over the *cagA* gene (Bodo Linz, personal communication), suggesting that this strain indeed contains a single copy of *cagA* despite having a genomic rearrangement otherwise similar to that of SS1/PMSS1.

In summary, we report here the sequences of two highly important related *H. pylori* strains. We provide insights into the variability within and between them and highlight the conclusion that a single fixed genome would not accurately represent these strains. One of the most striking findings from our genome analysis is the discovery of the unprecedented variation in *cagA* copy number, a discovery that we made by first analyzing the read depth over the *cagA* gene. Because *H. pylori* is not the only bacterial pathogen known to have a high rate of genomic plasticity, we suspect that similar efforts could reveal mutability in other bacterial genomes. This analysis could aid research into pathogenicity and outbreak tracing, which frequently rely on differential analysis of genomes under the assumption that the genomes for each isolate are relatively static and representative of the immediate population from which it was isolated. We suggest that future sequencing efforts include analysis of variation as part of the annotation and thus move a step closer to reporting the true sequences of the genomes under study.

## MATERIALS AND METHODS

### *H. pylori* strains and growth conditions.

A stock culture of *H. pylori* strain SS1 was provided by Adrian Lee and Jani O’Rourke (University of New South Wales, Australia) as a low *in vitro* subculture isolate from the initial SS1 isolate (18). SS1 was grown on Columbia horse blood agar (CHBA) plates, which contain Columbia agar (BBL) supplemented with 5% defibrinated horse blood, 5 mg trimethoprim/ml, 8 mg amphotericin B/ml, 10 mg vancomycin/ml, 50 mg cycloheximide/ml, 5 mg cefsulodin/ml, 2.5 U polymyxin B/ml, and 0.2% (wt/vol) beta-cyclodextrin. Plates were incubated under microaerobic conditions in a 37°C incubator with a gas mixture of approximately 7% O_2_ and 10% CO_2_, with the balance composed of N_2_.

A stock culture of *H. pylori* strain PMSS1 was provided by Manuel Amieva (Stanford University), who had obtained it from Adrian Lee (University of New South Wales, Australia). The provided culture was ~5 *in vitro* subcultures from the original. PMSS1 was grown on brucella agar plates containing 5% newborn calf serum and TVPA (trimethoprim, 5 mg/liter; vancomycin, 10 mg/liter; polymyxin B, 2.5 IU/liter; amphotericin B, 2.5 mg/liter) antibiotics. Incubation was carried out at 37°C under microaerophilic conditions of 5% O_2_ with the use of an Anoxomat system (Advanced Instruments, Norwood, MA).

To create freezer stocks, the cells were scraped off the respective plates; resuspended in a mixture of brucella broth, 10% fetal bovine serum (FBS), 25% glycerol, and 5% dimethyl sulfoxide; and frozen at −80°C. To resuscitate freezer stocks, a small quantity of frozen stock was placed onto a fresh plate and incubated as described above.

Deletion of *cagA* in PMSS1 was performed by insertion of a *CAT_rpsL* antibiotic resistance cassette to replace the entire *cagA* locus from 590 bp upstream of *cagA1* to 168 bp downstream of *cagA4*, using methods described previously (22).

### Isolation of genomic DNA from the working stock population for sequencing and PCR analysis.

To generate genomic DNA (gDNA) for sequencing and PCR, *H. pylori* strains were revived from the working freezer stock in the laboratory, placed onto the respective plates, and grown as lawns for 2 to 3 days as described above before being subcultured to a new plate to amplify the bacterial numbers. After four such subcultures to new plates, genomic DNA was isolated from *H. pylori* combined from five (SS1) or four (PMSS1) plates. For SS1, a Qiagen DNeasy kit (Qiagen, Valencia, CA) was used according to the manufacturer’s instructions. For PMSS1, bacterial cells were lysed with lysozyme and EDTA, and genomic DNA was purified using a QIAamp DNA minikit (Qiagen, Valencia, CA) according to the manufacturer’s instructions. The purity and integrity of the genomic DNA were assessed on a 2200 TapeStation with Genomic DNA ScreenTape (Agilent Technologies, Santa Clara, CA). For SS1, the sequenced bacterial population derived from bacteria that were 10 to 11 subcultures from the last single-colony purification step, and the PMSS1 bacteria were ~9 subcultures from the last single-colony purification step, where each subculture refers to moving a portion of the population from one plate to a new plate, to amplify bacterial numbers.

### SS1 genome sequencing.

Genomic DNA isolated from *H. pylori* SS1 was sequenced at the Genome Sequencing Center of the University of California, Santa Cruz (UCSC), in 2009, using SOLiD and 454 Titanium GS-FLX instruments. All library preparation and sequencing reactions were performed at this center.

The 454 library was sheared and size-selected to 400 to 900 bp according to the manufacturer’s protocol using a Lib-L library kit, checked for quality on a BioAnalyzer (Agilent), and sequenced according to Roche 454 titanium GS-FLX protocols on a full plate. This resulted in 557,959 reads passing the filters, with a mean length of 370 ± 136 bp and a 454 quality score of 29 ± 9.2; this represents roughly 128-fold coverage of the SS1 genome.

The SOLiD mate pair library was prepared using a SOLiD mate-paired library kit (ABI) on SS1 genomic DNA sheared and size selected to 2 to 3 kb. The library was quality-checked on a BioAnalyzer, subjected to emulsion PCR, and sequenced on 1 partition of a 4-partition slide on a SOLiD System 2.0 analyzer, using the mate pair sequencing protocol. This resulted in over 38 million “useable” read pairs. These reads were 25 bp in length, representing nearly 2 billion bp and over 1,000-fold coverage of the SS1 genome.

### SS1 *cagY* sequencing.

The SS1 *cagY* gene could not be assembled from the short-read data and was initially sequenced with C1 chemistry PacBio reads of an amplicon of the whole gene (for the primers used, see Table S4). The reads were aligned and a rough consensus sequence was formed from BLASR alignments (46). The rough consensus was fixed by hand, changing homopolymer lengths to make a single long ORF and to get better compatibility with the 454 and SOLiD data. The resulting sequence was used to build a hidden Markov model (HMM), which was then refined by model surgery using the SAM HMM package from UCSC (47), with training using a subset of the PacBIO reads. Subsequent PacBio sequencing using C2 chemistry run in 2012 could be assembled using HMMs without hand editing or 454 data and produced the same sequence. The *cagY* sequence includes 32 copies of the usual repetitive motif (48).

10.1128/mBio.02321-16.7 TABLE S4  Primers used in this work. Download TABLE S4, DOCX file, 0.5 MB. Copyright © 2017 Draper et al. 2017 Draper et al. This content is distributed under the terms of the Creative Commons Attribution 4.0 International license.

### PMSS1 genome sequencing.

PMSS1 was sequenced within the 100 K Pathogen Genome Project at the University of California, Davis (UC Davis). PacBio libraries were prepared as described previously (49). Briefly, 10 µg of gDNA that met size and quantity standards was fragmented using a Covaris g-Tube (Covaris, Woburn, MA) (50), normalized to between 1 and 5 µg, and used to construct sequencing libraries using a SMRTbell 10-kb library preparation kit (Pacific Biosciences, Menlo Park, CA). The final library submitted for sequencing was >9 kb and >25 ng/µl. Sequencing was performed at the UC Davis Genome Center using the PacBio RSII sequencing platform with C2 chemistry on a single flow cell following the instructions of the manufacturer (Pacific Biosciences) and as previously described (51).

For follow-up Illumina/HiSeq sequencing, isolated gDNA was sheared using a Covaris E220 instrument with a 96-microtube plate (Covaris Inc., Woburn, MA). Libraries were made using a Kapa HTP library preparation kit (KR0426, v3.13; Kapa Biosystems, Wilmington, MA) with dual-SPRI size selection. Libraries were constructed using an Agilent Bravo platform (Agilent Technologies, Santa Clara, CA). Library quantitation was done using Kapa SYBR fast quantitative PCR (qPCR) kits (Kapa Biosystems) to ensure a starting concentration of 400 ng and a fragment insertion size of between 350 and 450 bp (51). Libraries were indexed using Weimer 384 TS-LT DNA barcodes (Integrated DNA Technologies, Inc.). Sequencing was done at the UC Davis Genome Center (Davis, CA) on a HiSeq 3000 instrument using a paired-end 150-bp protocol (Illumina Inc., San Diego, CA).

### SS1 assembly: preprocessing.

The sequence data from the 454 run was subjected to a filtering step to remove reads matching known laboratory contaminants and the pHPS1 plasmid using Roche’s GS Reference Mapper (Newbler, version 2.0.01; now called gsMapper) software. The initial *de novo* assembly was performed using Roche’s *de novo* assembler (Newbler, version 2.0.01; now called gsAssembler [52]), using default settings except for changing the expected depth to 100. This initial assembly resulted in 47 contigs of length 97 to 181,383 bp. A later *de novo* assembly that also excluded the IS*607* reads was used for the main scaffolding (51 contigs, 111 to 181,591 bp).

Sequence data from the SOLiD run were interleaved using ABI’s abiDeNovo^239,164^ preprocessor script to generate colorspace (.csfasta) format reads. The SOLiD colorspace reads were mapped to the contigs to determine adjacencies, using custom Python programs that operated in colorspace. For reads mapping within a contig, the mean length of the mate pair was 2,053 bp, with a standard deviation of 399 bp. After rejection of about 18% of SOLiD mate pairs with high coverage to avoid spurious matches [identified as degenerate sequences such as poly(AT) and a contaminant from an externally supplied reagent], only about 27% of the pairs mapped uniquely to a single contig, 37% of the pairs mapped to multiple locations, and 18% of the pairs connected two contigs uniquely.

### SS1 assembly: IS*607*.

Due to the simultaneous presence and absence of IS*607* at multiple sites in the genome causing many contig break points and ambiguous scaffolding graphs, IS*607* was initially pulled out and assembled separately. Reads mapping partially or completely to IS*607* were separated from the rest of the reads and were assembled in two ways: by a reference-based assembly mapping to previously published IS*607* sequences and by *de novo* assembly. The two methods produced identical sequences. The coverage of the IS*607* contig was about four times higher than the average genomic coverage, and the *de novo* assembly had 8 additional contigs, implying the presence of 4 insertion sites for IS*607*.

### SS1 assembly: genome closure.

The contigs of the *de novo* assembly of 454 reads (excluding reads mapping partially or fully to pHPS1 or IS*607*) were scaffolded by hand with the aid of custom Python scripts using the SOLiD mate pairs that joined two contigs. Contigs that had high read coverage and ambiguous neighbors were used repeatedly in the scaffolding, under the assumption that the sequence occurred multiple times in the genome. This initial scaffolding process resulted in 7 scaffolds.

A mapping assembly of the 454 reads was done using the scaffolds (plus pHPS1, separately assembled) as a reference, resulting in 22 contigs that did not fully cover the genome. Reads that did not match completely were assembled *de novo*, adding another 43 contigs. SOLiD mate pairs were mapped to these contigs to determine the ordering and orientation of the larger contigs, with shorter ones placed by alignment to the earlier scaffolds. This resulted in a complete chromosome, minus the IS*607* insertions.

A mapping assembly was done of the 454 reads onto the putative chromosome, pHPS1, and IS*607*. Chimeric reads between the chromosome and the IS*607* contig identified four insertion sites for IS*607*, and the SOLiD mate pairs were used to check these insertions. Three of them were strongly supported by the SOLiD mate pairs, but the third had strong evidence for both insertion at the site and no insertion at the site.

SOLiD mate pairs were used to test possible inversions (suggested by complementary matching sequences at each end of the putative inversion) and to test various SNPs suggested by variations in the 454 assemblies. The genome was polished by doing several rounds of 454 mapping assembly and checking proposed changes with the SOLiD mate pairs, but the *cagY* and *cagA* regions remained problematic and had to be tackled separately as described in the “*cagY*” and “*cagA*” sections.

### SS1 *cagA* assembly.

The *cagA* region was assembled separately from the rest of the genome by selecting all 454 reads that mapped to *cagA* contigs or the surrounding region and assembling them *de novo* with Newbler. The reads were supplemented by Sanger reads, with primers selected so that the Sanger reads could help with scaffolding (Table S4). As with the whole-genome assembly, the SOLiD reads were used to help find SNPs, to check the scaffolding, and to suggest the number of repeats. Based on the coverage seen with the SOLiD mate pairs and 454 reads, we conjectured the presence of three to four copies of the *cagA* gene, which was confirmed by Southern blotting. The short-read data (even the Sanger sequences) did not allow us to distinguish any differences between the repeats.

### *cagA* copy number verification and CagA expression analysis.

To determine the copy number of *cagA* in the SS1 and PMSS1 genomes, a Southern blot was performed. Genomic DNA was digested with SspI-HF (New England Biolabs) for 2 h. Digested DNA was separated on a 0.5% agarose gel overnight at 0.75 V/cm and transferred to a nylon membrane. A fragment of *cagA* was PCR amplified from SS1 (bp 1217 to 1514) with primers D008 and R008 (Table S4) and labeled with biotin using a North2South biotin Random Prime Labeling kit (Thermo Scientific). Hybridization and detection were carried out with a North2South chemiluminescent hybridization and detection kit according to the manufacturer’s instructions. *cagA* copy numbers were determined based on fragment size and the known restriction map of the* cagA* locus (Fig. 4A).

For Western blot analysis, bacterial lysates of PMSS1 single-colony isolates containing 4, 2, or 1 copies of *cagA* were prepared by growth in liquid culture to mid-exponential phase followed by sonication on ice. Lysates were quantified by Bradford assay, and 10 µg each was loaded onto a 7.5% Mini-Protean TGX precast gel (Bio-Rad Laboratories). Separated proteins were transferred to a polyvinylidene difluoride (PVDF) membrane which was then incubated with rabbit anti-CagA (Austral Biologicals) followed by peroxidase-labeled anti-rabbit IgG (GE Healthcare). To normalize loading, the blot was also incubated with anti-UreB (LifeSpan BioSciences). Enhanced chemiluminescence (ECL) reagents were utilized for visualization of bound antibody (Thermo Scientific).

### PMSS1 PacBio assembly.

PMSS1 was assembled* de novo* using the PacBio SMRT Analysis portal (version 1.3), consisting of the assembly by hierarchical genome assembly process (HGAP) and polishing with Quiver (53). This produced a complete linear PMSS1 genome that began and ended with imperfectly assembled copies of the *cagA* repeats. Closure of this gap was performed by mapping the PMSS1 *cagA* repeat region reads to the SS1 genome. There were no reads that spanned a section from a location before the *cagA* genes to one after the *cagA* genes, so the number of copies could not be conclusively determined from the PacBio data. Coverage levels suggested either three or four copies. We decided to include four copies, based on the coverage and the Southern blot data.

### Assembly: plasmid pHPS1.

For SS1, assembly of the plasmid pHPS1 was performed by mapping the 454 data to the published pHPS1 sequence (24); the recovered sequence was identical to the published plasmid except for a deletion of a single T at position 3274, adjacent to the R3 repeat region. Roughly 56,000 454 reads matched pHPS1, representing roughly 1,000× coverage of the 5.8 kb plasmid and ~10% of the entire 454 run. As the 5.8-kb plasmid is only ~0.3% the size of the 1.5 Mb of the genome, this is a disproportionate number of reads. The reason for this is unclear, but it is possible that this is a moderately high-copy-number plasmid (~30 to 100 copies).

For PMSS1, the *de novo* assembly had a unitig that appeared to be 2.46 copies of pHPS1 assembled as a tandem repeat rather than as a circular molecule. Mapping the reads to the unitig revealed a region that had higher coverage, with boundaries that corresponded to the best copy of the pHPS1 sequence. This region was extracted from the unitig, with boundaries chosen so that the sequence could be circularized to correspond to the unrolled sequence in the *de novo* assembly. Remapping reads to this shorter sequence successfully mapped 16,588 reads to the shortened plasmid sequence, versus 16,647 for the unrolled sequence in the *de novo* assembly. This is close enough that there is no justification for the unrolling. Coverage of the plasmid was about 9,100×.

The PMSS1 sequence has exactly the same deletion of T as the SS1 plasmid sequence, relative to the published pHPS1 sequence (24). There is also one insertion of 213 bases relative to the published pHPS1 sequence, overlapping the R2 repeat region, but we did not pursue this difference.

### Corrections to genomes.

To confirm the PMSS1 PacBio assembly sequence, all differences between SS1 and PMSS1 were compared with the results of HiSeq sequencing of four other PMSS1 isolates that were obtained after independent 8-week mouse infections (J. V. Solnick and L. M. Hansen, unpublished observations). The PMSS1 assembly was corrected to the SS1 sequence if the HiSeq data for all 4 other PMSS1 sequences agreed with the SS1 sequence, since it seemed unlikely that the identical changes would occur during independent mouse infections of PMSS1. However, one gene (HPYLPMSS1_01047) displayed a cluster of SNPs where the SS1 version was found in 3/4 isolates; the PMSS1 sequence was corrected to match SS1 for this gene, but a note about this finding was added to the gene’s annotation. For SS1, the SOLiD data were used to check whether any of the differences reflected potential sequencing errors in the SS1 sequence. In addition, one variable SNP in the *flhB* gene was corrected and confirmed based on Sanger sequencing. Sanger sequencing was also used to confirm the sequences around the large SS1 inversion site.

Two regions of the initial SS1 assembly that differed from PMSS1 were found to have been misassembled in SS1. One region of the SS1 sequence for which the Newbler assembler had reported a high-confidence structural variation appeared to be a tandem repeat of 21 bases that was a little too long to resolve with 454 reads. This region was copied from the PMSS1 genome, where it had 12 copies. The other SS1 misassembly also occurred in the SS1 genome in a duplicated region, near the inverted repeat that marked the large inversion between PMSS1 and SS1. The initial 454 assembly had assigned different reads to the two regions, but the two regions were identical in PMSS1, and we confirmed with Sanger sequencing that they were identical in SS1 also.

### Genome annotation.

The SS1 genome was annotated using Prokka v 1.11 (54), the RAST server (55), and NCBI Prokaryotic Genome Annotation Pipeline v 2.7 (56). Annotations were compared by hand within the program Geneious (v 5.6–v 9.1) (57). In most cases, the Prokka annotation was retained; where annotations differed significantly, each ORF was checked by hand against other *H. pylori* genomes and protein databases and was typically assigned the most generic annotation call. Genes of particular interest to our laboratories, such as the *cag* PAI genes, were annotated by hand. Where genes appeared to be obviously disrupted by frameshifts, the “complete” ORFs were annotated in the genes track as pseudogenes. Annotation of PMSS1 was performed by transferring the annotation from SS1 to the PMSS1 sequence in Geneious and reannotating pseudogenes. Locus tag numbers were assigned to SS1 according to the PMSS1 (ancestral) inversion orientation to ensure correspondence of gene numbering between the two genomes.

### Isolation of single colonies for variation analysis.

Single colonies were isolated from the SS1 or PMSS1 freezer stock cultures described in the “*H. pylori* strains” section. An aliquot from the freezer stock was struck onto solid media to obtain single colonies. Each colony was then restruck to obtain a greater number of bacteria, and then genomic DNA was prepared as described in the “Isolation of genomic DNA” section.

### Transposon IS*607* prevalence and location verification.

To verify the IS*607* insertion sites observed in the SS1 sequence data, PCR amplification was performed using primers listed in Table S4 and products for all four sites were confirmed by Sanger sequencing.

### SS1 and PMSS1 large-inversion verification.

To verify the orientation of the region that was inverted between SS1 and PMSS1, we designed primer pairs that would generate PCR products spanning the inverted region boundaries (Table S4 and Fig. 3). PCR products were analyzed by gel electrophoresis and by DNA sequencing with primers that annealed within the PCR products (Table S4).

### Core and cloud genome analysis.

The predicted *H. pylori* SS1 genes were compared to those of a set of 41 *H. pylori* genomes spanning six *H. pylori* phylotypes that were downloaded from PATRIC (http://patricbrc.vbi.vt.edu/portal/portal/patric/Home) and the NCBI website (http://www.ncbi.nlm.nih.gov/genome/browse/) (25). This set of genomes harbors 949 genes that are present in ≥95% of them and thus can be considered “core genes.” SS1 was also analyzed for genes that are present in fewer than 15% of genomes. For both analyses, SS1 data were compared using both Roary (26) and ProteinOrtho (58). Each predicted missing core gene or present cloud gene was examined individually to confirm its status. For the cloud genes, each gene was compared using tBLASTn to *H. pylori* TaxID 210, which contained 160 complete *H. pylori* genomes. Positive matches were counted as those with an E value of less than 10^−10^, with coverage over 75% and without significant gaps. Each cloud gene was further examined using PSI-BLAST, Pfam, and PHYRE to check for informative homology.

### Accession number(s).

The GenBank accession number for the *H. pylori* strain SS1 chromosome is CP009259, and that for its plasmid pHPYLSS1 is CP009260; the GenBank accession number for the PMSS1 chromosome is CP018823, and that for its plasmid pHPYLPMSS1 is CP018824. The *IS*607 sequence is available as GenBank accession number KY555128. The GenBank BioSample numbers for SS1 and PMSS1 are SAMN03331743 and SAMN04362855, respectively. The GenBank BioProject numbers for this work are PRJNA256258 (SS1 sequencing and assembly), PRJNA203445 (100K Pathogen Genome Project, PMSS1 sequencing), and PRJNA306775 (PMSS1 assembly and annotation). Raw read data are available on the NCBI Sequence Read Archive (SRA) under accession numbers SRR5236049 (PMSS1) and SRP099088 (SS1).
