# Supplementary material for: Fallacy of the Unique Genome: Sequence Diversity within Single Helicobacter pylori Strains
Source: mBio. 2017 Feb 21;8(1):e02321-16. doi: 10.1128/mBio.02321-16 (PMC5358919; doi:10.1128/mBio.02321-16)
Supplement: TABLE S1 [file mbo001173212st1.docx]

| **Putative missing protein** | **Outcome** | **Description** |
| --- | --- | --- |
| L-lactate permease (*lldP*) | Disrupted in SS1 (HPYLSS1_00131) but not PMSS1 | SS1 ORF has a frameshift resulting in the encoded protein being truncated by ~ 25%. Alteration is in a homopolymeric repeat. *H. pylori* strains encode two lactate permeases with adjacent genes. |
| Carbon starvation protein A (*cstA*) | Disrupted in SS1 but not PMSS1 | SS1 ORF has a frameshift resulting in it being split. Alteration is not in a homopolymeric repeat. The “CstA” domain is mostly within the N terminal portion so may retain function. |

Supplemental Table 1: Core genes that are missing in the SS1 genome.
