# Supplementary material for: Fallacy of the Unique Genome: Sequence Diversity within Single Helicobacter pylori Strains
Source: mBio. 2017 Feb 21;8(1):e02321-16. doi: 10.1128/mBio.02321-16 (PMC5358919; doi:10.1128/mBio.02321-16)
Supplement: TABLE S2 [file mbo001173212st2.docx]

| Gene Name | Size (AA) | | Annotation/Function | | Prevalence^1^ | | |
| --- | --- | --- | --- | --- | --- | --- | --- |
| HPYLSS1_00046 | | 157 | | Hypothetical, with possible sugar or peptidoglycan binding protein. | | 4% |  |
| HPYLSS1_00455 | | 34 | | Hypothetical | | 3% |  |
| HPYLSS1_00486 | | 425 | | IS607 putative transposase | | 11% |  |
| HPYLSS1_00573 | | 199 | | NYN/LabA-domain, unknown function. | | 3%^2^ |  |
| HPYLSS1_00581 | | 47 | | Hypothetical | | 13% |  |
| HPYLSS1_00963 | | 111 | | Hypothetical. Encoded in plasticity zone | | 1% |  |
| HPYLSS1_00960 | | 233 | | Hypothetical. Encoded in plasticity zone | | 6% |  |
| HPYLSS1_00740 | | 395 | | Hypothetical with SMC domain | | 4% |  |
| HPYLSS1_00731 | | 63 | | Hypothetical | | 3% |  |
| HPYLSS1_01225 | | 51 | | Hypothetical. 1236-137-1238 form operon. | | 1.88%* |  |
| HPYLSS1_01226 | | 51 | | Hypothetical. | | 1.88%* |  |
| HPYLSS1_01227 | | 228 | | Hypothetical. COG4278 domain (unknown function) | | 1.88%* |  |
| HPYLSS1_01357 | | 72 | | His- and Glu-rich Hpn-like protein | | 9.38% |  |
| Plasmid pHPYLSS1 | |  | |  | | 5.0% |  |
|  | |  | |  | |  |  |
| Pseudogenes |  |  | |  | |  |  |
| HPYLSS1_00606 | | 146 | | Hypothetical | | 1% |  |
| HPYLSS1_00961 | | 210 | | Hypothetical. Encoded in plasticity zone | | 6% |  |
| HPYLSS1_01442 | | 207 | | Two ORFs in SS1; Found as one ORF in most *H. pylori*. Hypothetical | | 14.4% |  |
|  | |  | |  | |  |  |

Supplemental Table 2. Putative cloud genes of SS1.

Notes as follows. 1: Prevalence is defined as the number of genomes that contain a particular gene within the *H. pylori* TaxID210 of NCBI. Within TaxID210, there were 160 genomes that were scaffold or complete as of February 2016, so this number is used as the denominator. For example, if a gene was found in two genomes, 2/160 = 1.25%. * Found as operon in *H. pylori* strains HUP-B14, SJM180, and SouthAfrica20. Putative functions were determined using a combination Psi-BLAST, Pfam, and/or Phyre. The cloud genes listed as "pseudogenes" contain alterations in polynucleotide tracts that interrupt the entire expected gene, either truncating it or splitting it into two predicted ORFs. These pseudogenes were similarly disrupted in PMSS1.
