# Supplementary figures and images for: Fallacy of the Unique Genome: Sequence Diversity within Single Helicobacter pylori Strains
Source: mBio. 2017 Feb 21;8(1):e02321-16. doi: 10.1128/mBio.02321-16 (PMC5358919; doi:10.1128/mBio.02321-16)

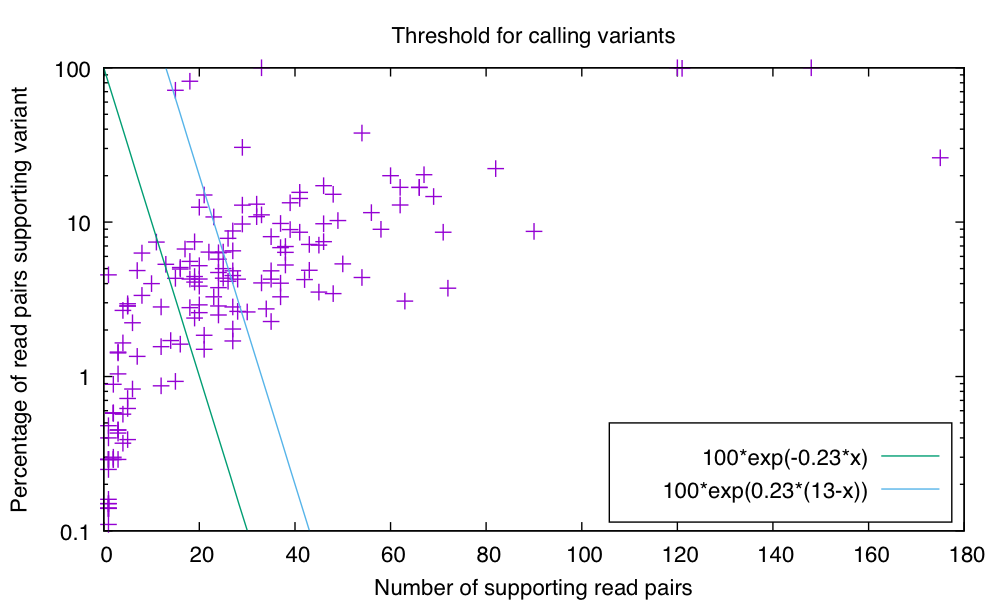

Supplement: FIG S1 [file mbo001173212sf1.tif]

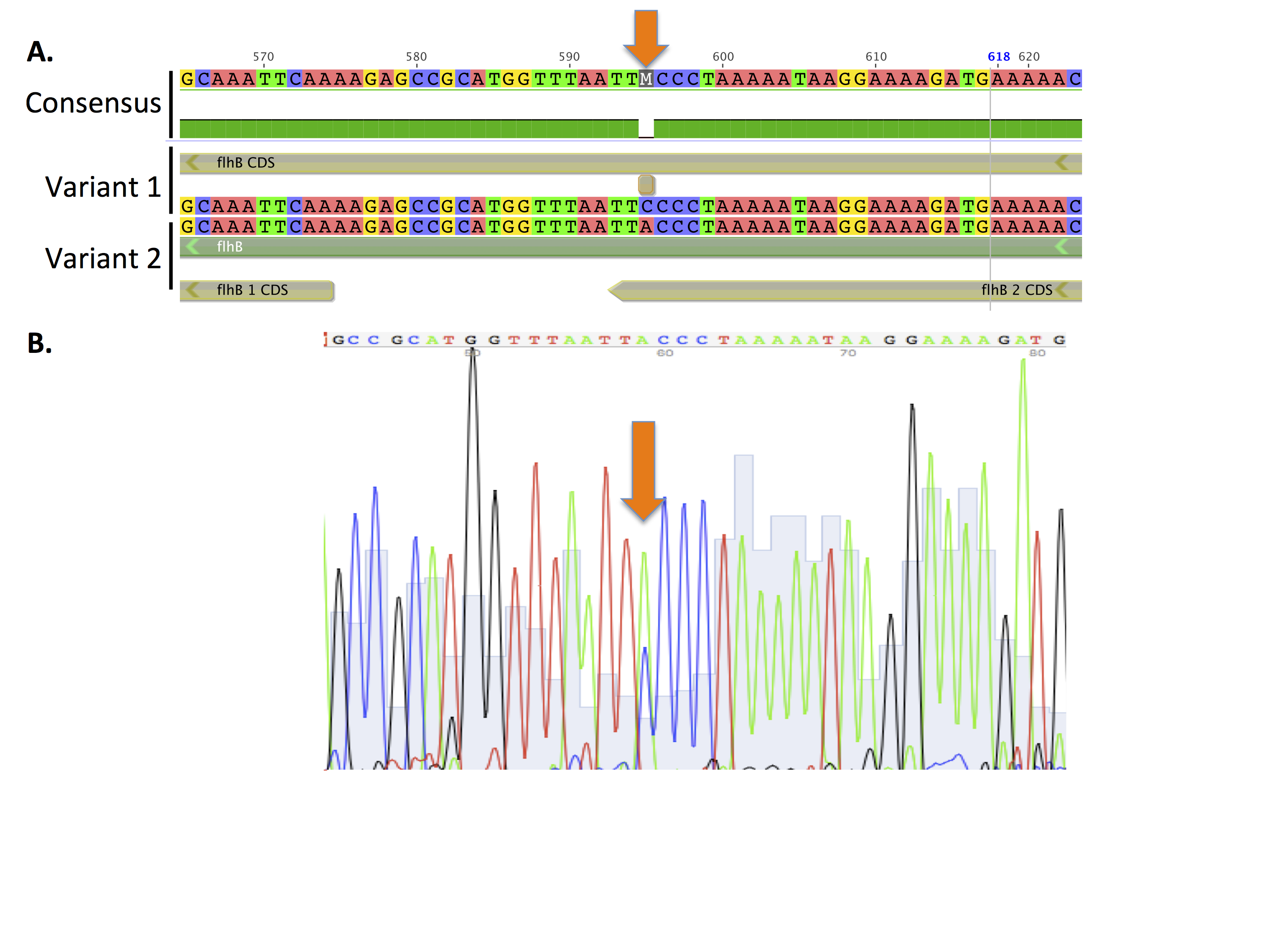

Supplement: FIG S2 [file mbo001173212sf2.tif]

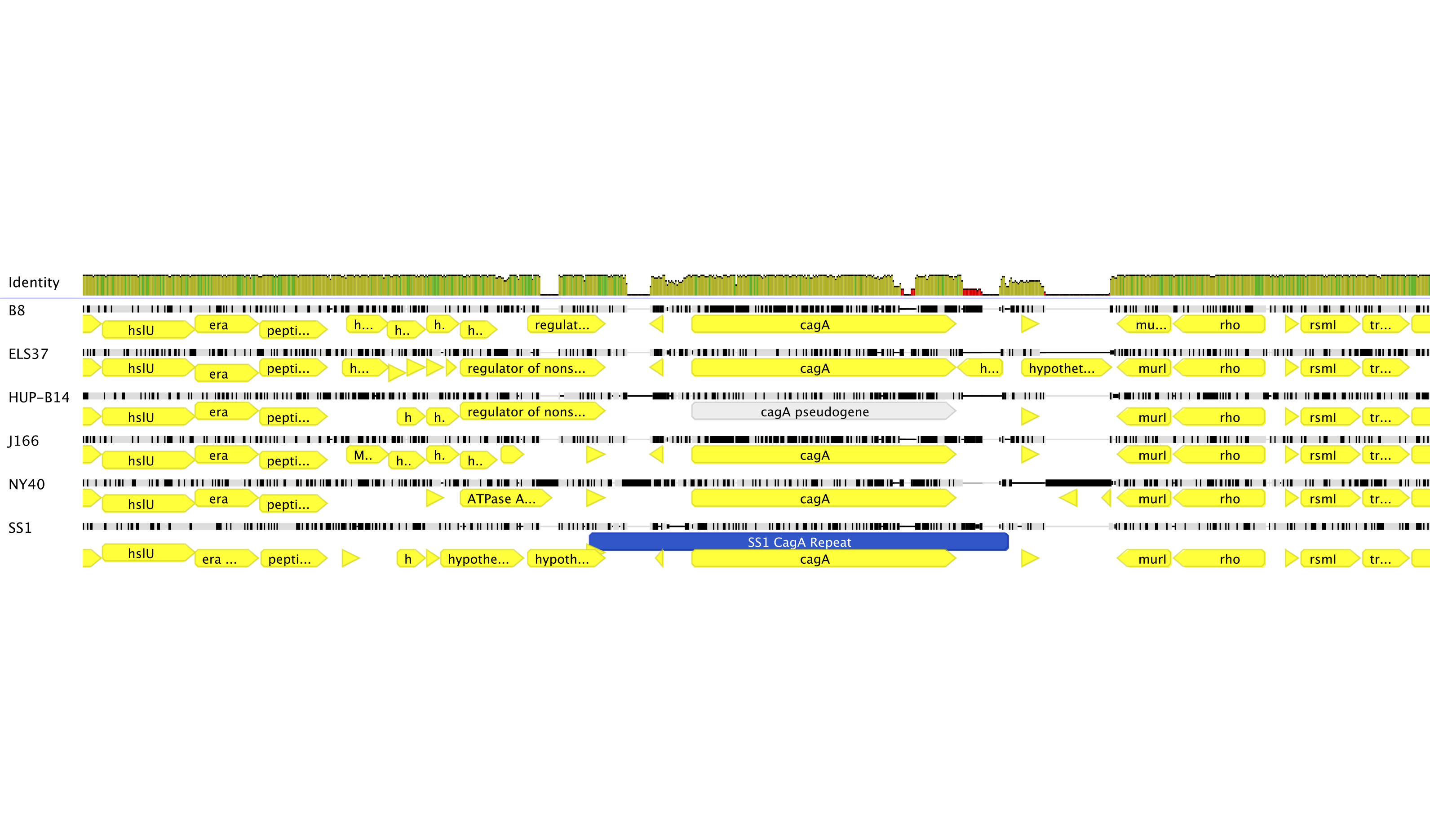

Supplement: FIG S3 [file mbo001173212sf3.tif]
