# Supplementary material for: Fallacy of the Unique Genome: Sequence Diversity within Single Helicobacter pylori Strains
Source: mBio. 2017 Feb 21;8(1):e02321-16. doi: 10.1128/mBio.02321-16 (PMC5358919; doi:10.1128/mBio.02321-16)
Supplement: TABLE S3 [file mbo001173212st3.docx]

| **Start (PMSS1)** | **Difference** | **Homopolymer?** |
| --- | --- | --- |
| 25349 | SS1:PMSS1 Difference::del:A | A |
| 102852 | SS1:PMSS1 Difference::C:del | C |
| 116555 | SS1:PMSS1 Difference::CTG:ATT | T |
| 116594 | SS1:PMSS1 Difference::GAGCGCC:TAACGCT | No |
| 204105 | SS1:PMSS1 Difference::2del:2T | T |
| 227039 | SS1:PMSS1 Difference::del:A | A |
| 305992 | SS1:PMSS1 Difference::del:T | T |
| 322837 | SS1:PMSS1 Difference::del:T | T |
| 343490 | SS1:PMSS1 Difference::del:A | A |
| 538774 | SS1:PMSS1 Difference::2del:AA | A |
| 632168 | SS1:PMSS1 Difference::del:A | A |
| 824739 | SS1:PMSS1 Difference::G:del | G |
| 885399 | SS1:PMSS1 Difference::del:T | T |
| 1271357 | SS1:PMSS1 Difference::del:A | A |
| 1398486 | SS1:PMSS1 Difference::2del:2G | G |
| 1551191 | SS1:PMSS1 Difference::2del:2T | T |
| 1555134 | SS1:PMSS1 Difference::del:A | A |
| 5172 (plasmid) | 213bp insertion in PMSS1 plasmid | No |

**Supplemental Table 3. SS1-PMSS1 differences that were in intergenic regions.**

Start indicates the base position in PMSS1. Differences are given as SS1 compared to PMSS1, with the SS1 sequence listed first. Homopolymer indicates whether the difference was in a homopolymer tract of length 3 or more, and the base identity.
