# Supplementary material for: Fallacy of the Unique Genome: Sequence Diversity within Single Helicobacter pylori Strains
Source: mBio. 2017 Feb 21;8(1):e02321-16. doi: 10.1128/mBio.02321-16 (PMC5358919; doi:10.1128/mBio.02321-16)
Supplement: TABLE S4 [file mbo001173212st4.docx]

**Supplemental Table 4: Primers used in this work**

| **Primer name** | **Primer sequence** | **Target** |
| --- | --- | --- |
| is607F | TTTACGCCTATGGACTTGACG | facing outwards from IS607 *orfB* |
| is607R | CTAAAAGCTTACTCGCTTGACC | facing outwards from IS607 *orfA* |
| is1f | AAGGCACGATGGAGTGGAGC | IS607 insertion site 1 |
| is1r | TCGCCAAAGCGTCTTCATGG | IS607 insertion site 1 |
| is2f | CTAAAATCCTTCAAGCCATAGG | IS607 insertion site 2 |
| is2r | TTCTTTAGTGTCTTCTTGCTCC | IS607 insertion site 2 |
| is3f | TCATGATAGCCAATAAAGTAGG | IS607 insertion site 3 |
| is3r | TCGTGCCTAGAACCGATGG | IS607 insertion site 3 |
| is4f | TTTAGTCTATGGGGTTGCTGG | IS607 insertion site 4 |
| is4r | TTAAGAGCAAGTTCCCTAAAGC | IS607 insertion site 4 |
| A-SS1inv | GTTTTTAGTTTAGCGGCTACGC | PCR Amplification of large inversion |
| IR1-SS1inv | GCAGTTTAAGAAATTCTATTGAATGGC | PCR Amplification of large inversion |
| C-SS1inv | CTTACCGCTTCCTGTAACCC | PCR Amplification of large inversion |
| D-SS1inv | TTGAGGCTTATTTTTCTCACACCC | PCR Amplification of large inversion |
| IR2-SS1inv | ATGAGAGATTGAGAGGGATCGTT | PCR Amplification of large inversion |
| Z-SS1inv | CGCTATACTGAAACGCGCC | PCR Amplification of large inversion |
| IR_Seq1 | AGCTTAGGTTAGTCGTAGATG | Sequencing of region around large inversion |
| IR_Seq2 | ACGGACATGAACGAAGAG | Sequencing of region around large inversion |
| IR_Seq3 | TACCAAACCACGAGCGAGAG | Sequencing of region around large inversion |
| IR_Seq4 | ACGATACCGGAAGCGATAGAG | Sequencing of region around large inversion |
| IR_Seq5r | TAGCTTGCGTGATTGCTTC | Sequencing of region around large inversion |
| D008 | ATAATGCTAAATTAGACAACTTGAGCGA | Amplification of a portion of *cagA* for Southern blot probe |
| R008 | TTAGAATAATCAACAAACATCACGCCAT | Amplification of a portion of *cagA* for Southern blot probe |
| cagY_wF | 5’-[Phos]-TCCTCTAGAAGAGCCTAGTTG | cagY gene for PacBio sequencing |
| cagY_wR | 5’-[Phos]-AACTATGGTGAATTGGAGCG | cagY gene for PacBio sequencing |
| flhB-F1 | 5’AAA CAA GGC GAA GAA ATG AGC C | flhB amplification and sequencing primer |
| flhB-R1 | 5’CTC CTT GAT GGG AGC TTG ATC A | flhB amplification and sequencing primer |
